# Probing the atomistic origins of magnetic heating with colloidal solutions of ferrimagnets

**DOI:** 10.1063/5.0344785

**Published:** 2026-09

**Authors:** Noah Kent, Keisuke Nagao, Daniel Suzuki, Byung Hun Lee, Nannette Chattman, Jorge Marquez Chavez, Katherine Lei, Ye Ji Kim, Jacob Beckham, Geoffrey Beach, Polina Anikeeva

**Affiliations:** 1 Research Laboratory of Electronics, Massachusetts Institute of Technology, Cambridge, Massachusetts 02139, USA; 2 McGovern Institute for Brain Research, Massachusetts Institute of Technology, Cambridge, Massachusetts 02139, USA; 3 Department of Materials Science and Engineering, Massachusetts Institute of Technology, Cambridge, Massachusetts 02139, USA; 4 K. Lisa Yang and Hock E. Tan Center for Molecular Therapeutics in Neuroscience, Massachusetts Institute of Technology, Cambridge, Massachusetts 02139, USA; 5 Department of Brain and Cognitive Sciences, Massachusetts Institute of Technology, Cambridge, Massachusetts 02139, USA

## Abstract

Understanding the physics of alternating magnetic field-induced heating in magnetic materials is important for optimizing the performance of systems, such as electrical transformers, spintronic devices, and nanomaterials used for controlling biological signaling and cancer ablation. Heating in these systems can be accurately explained by theories where magnetization of the material is uniform and the only relevant energy barrier is magnetic anisotropy. In these models, total heating is proportional to hysteresis loop area and scales with the total magnetization. However, these models assume uniform magnetization, which does not necessarily hold in nanostructured materials. A more accurate model should consider all nanomagnetic energy dynamics and show that heating is a result of the rapid movement of individual spins. Here, colloidal solutions of near-compensation GdCo ferrimagnets are used to probe the atomistic energetic losses that occur in non-uniform magnetic systems. Although near-compensation ferrimagnets should not heat according to uniform-magnetization-based theories, we find that heating occurs in a manner that is not dependent on the total hysteresis loop area, but rather on the movement of spins in the Gd and Co sublattices. The atomistic magnetic spin dynamics in ferrimagnets can be connected to uniform-magnetization-based heating theories by considering the energetic losses of the individual sublattices. This modeling predicts that magnetic field-induced heating is optimized in systems where exchange-driven dynamics drive rapid movement of spins, an insight that could allow for optimization of alternate magnetic field induced heating in systems with a non-uniform magnetization.

## I. INTRODUCTION

Heat induced by alternating magnetic fields (AMFs) in magnetic materials influences the performance of technologies ranging from large-scale power generation^1,2^ to nanoscale data manipulation.^3,4^ For example, excess heat generated in electrical transformers reduces power conversion efficiency^2^ and can thermally disrupt the nanoscale magnetic order in hard drives,^4^ corrupting data. In contrast, heat induced by AMFs in magnetic materials is beneficial for some biological applications, including hyperthermia cancer therapy^5^ and magnetic control of thermally sensitive biological signaling.^6^

Most biological tissues do not interact with or attenuate magnetic fields, allowing them to penetrate deep into living systems.^7^ To convert an external magnetic field into a stimulus capable of activating a biological cascade, a nanomagnetic transducer is needed. To date, nanomagnetic transducers have been developed to convert magnetic fields into physical force,^8,9^ chemical gradients,^10^ voltage,^11^ or heat.^6,7,12^ Optimizing the physics behind each of these transduction modalities results in more effective and targeted biological actuation at lower nanotransducer concentrations.

**FIG. 1.**
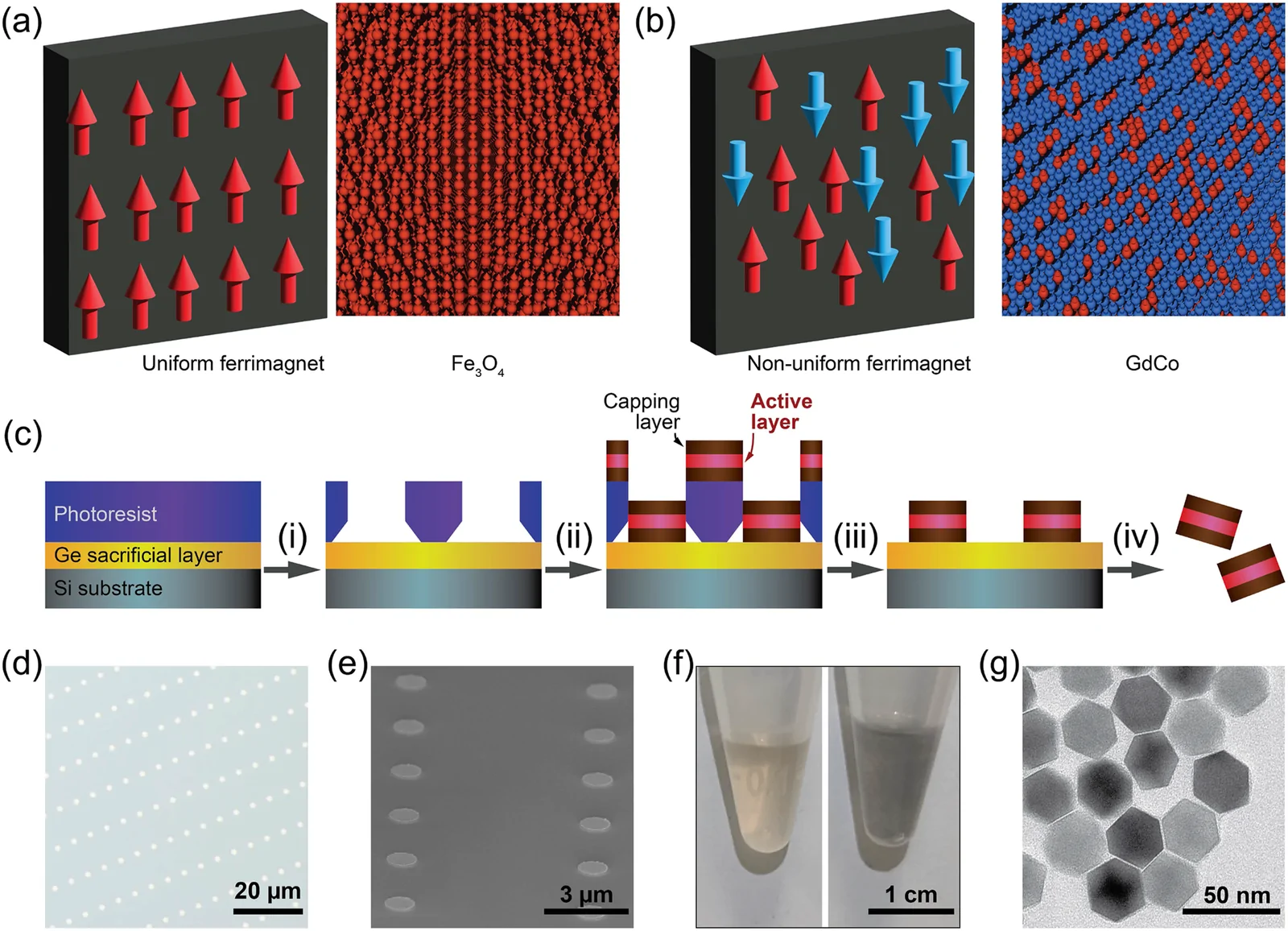
Schematic and atomistic description of magnetic samples. (a) Schematic of a uniformly magnetized system (left). Atomistic view of the active magnetic lattice in a 29 nm diameter magnetite (right). (b) Schematic description of a non-uniform ferrimagnetic system, where the different colors of arrows indicate different sub-lattices (left). Atomistic view of active magnetic lattices in a simulation of an amorphous GdCo ferrimagnetic film (right). (c) Modified liftoff procedure with sacrificial Ge layer to allow for release of particles into colloidal solution with the following steps: (i) exposure and development, (ii) deposition, (iii) liftoff, and (iv) sacrificial layer release. (d) Optical microscopy image of GdCo disks. (e) Tilted SEM image of GdCo disks. (f) Colloidal solution of a 29 nm diameter magnetite (left) and GdCo disks (right). (g) TEM image of faceted 29 nm diameter magnetite particles.

Understanding AMF heating dynamics at the nanoscale is particularly important for thermal nanotransducers or magnetic nanoparticles, which convert an AMF to heat. Thermal nanotransducers can be used to manipulate biological signaling by activating heat-sensitive receptors^6,12,13^ or to ablate cancerous cells via hyperthermia.^5^ These nanoparticles are often composed of a biocompatible magnetic material such as magnetite and are typically less than 50 nm in size.^6,7^ Based on these dimensions and depending on the material, they are usually approximated as macrospin (a single-domain magnetic nanoparticle)^14^ [Fig. 1(a)].

Theories typically describing AMF-induced heating^15,16^ consider systems where each nanoparticle can be described as having uniform magnetization [Fig. 1(a)]. However, in reality, many nanoscale magnetic systems exhibit non-uniform magnetization, such as magnetic vortex states, the multi-domain states found in hard drives, or Hopfions^8,11,17^ [Fig. 1(b)]. Understanding the response of non-uniform magnetic systems to an AMF demands an alternative approach that describes the fundamental dynamics of individual spins.

In this work, we probe the atomistic origins of AMF heating by comparing the heating in systems that possess uniform magnetization (i.e., single domain magnetite nanoparticle) with systems that have a non-uniform, but collinear, magnetic structure (i.e., near compensation ferrimagnets). We find that while the uniform-magnetization-based theory accurately explains the heating of a wide variety of magnetic systems,^2,15,16,18,19^ this theory underestimates the heating in the non-uniform magnets by nearly two orders of magnitude. By considering the energy loss due to atomistic spin dynamics, we are able to accurately predict heating even in this non-uniform system. Understanding the atomistic origins of nanoscale heating will allow for improved optimization of material response to AMFs across applications.

## II. EXPERIMENTAL RESULTS

To probe the atomistic origins of magnetically induced heating in nanomaterials, we experimentally studied induced heating in response to an AMF of two ferrimagnetic materials and a paramagnetic material. In a ferrimagnet, exchange coupling induces magnetic spins in subsets of atoms (sublattices) with a total non-zero system magnetization. Here, we consider two types of ferrimagnets with distinct coupling between sublattices, termed A and B. In the first type of ferrimagnet, A–A coupling is ferromagnetic, A–B coupling is antiferromagnetic, and B–B coupling is antiferromagnetic. Since B–B coupling is antiferromagnetic, sublattice B has an antiferromagnetic field response. In particular, below spin flop fields,^20^ sublattice B exhibits a negligible response to external fields and does not possess an open hysteresis loop.^20,21^ Hence, in these systems, sublattice A is the only sublattice that exhibits a ferromagnetic-like hysteresis loop. As a result, these systems can often be accurately described as possessing uniform magnetization [Fig. 1(a)], and we will refer to these systems as “uniform ferrimagnets.” In the other type of ferrimagnetic system considered here, B–B coupling is ferromagnetic instead of antiferromagnetic. In these systems, both sublattices A and B are responsive to external fields and exhibit ferromagnetic-like hysteresis loops [Figs. 1(b) and S2]. These systems will be referred to as “non-uniform ferrimagnets.”

Amorphous GdCo (a-GdCo) is an example of a non-uniform ferrimagnet composed of two ferromagnetic sublattices (Gd and Co) that are antiferromagnetically coupled to each other [Fig. 1(b)]. In a-GdCo, the Gd–Gd and Co–Co couplings are ferromagnetic and the Gd–Co coupling is antiferromagnetic.^22–24^ As a result, the two ferromagnetic sublattices are aligned antiparallel to each other, and because the Gd and Co atoms are randomly distributed in a-GdCo, the magnetic system is non-uniform on the atomic scale. In a-GdCo, the total magnetization can be tuned by changing the ratio of Gd to Co. At 22% Gd,^23^ the two sublattices’ magnetizations cancel each other out, such that the net magnetic moment is zero. GdCo ferrimagnets with an amount of Gd near 22%, but not exactly 22%, are considered to be a near-compensation ferrimagnet. Here, we prepared near-compensation amorphous Gd_25_Co_75_ ferrimagnetic disks (1.7 ± 0.1 *μ*m diameter; 30 thick) using standard photolithographic techniques [Figs. 1(c)–1(f), methods]. A colloidal solution of the a-GdCo disks was prepared by releasing the fabricated disks via dissolution of the sacrificial layer (Ge) [Fig. 1(c), methods]. This non-uniformly magnetized, but collinear, magnetic material is well-suited to evaluate theoretical models of magnetic heating.

As controls, two magnetic systems that can be accurately described as having a uniform magnetization were prepared: uniform ferrimagnetic particles and paramagnetic particles. A common AMF thermal nanotransducer, faceted magnetite nanoparticles (29 nm diameter) synthesized via a thermal decomposition method [Figs. 1(f) and 1(g), methods],^6,7^ served as a ferrimagnetic^20,21^ control, which can be approximated as having uniform magnetization.^25,26^ Magnetite nanoparticles are commonly used for hyperthermia and magnetic control of biological signaling.^5,6,12,13^ At room temperature, 29 nm magnetite nanoparticles can be described as single domain magnets^14,20,21^ and their heating should be accurately predicted using a theory that considers uniform magnetization. Palladium disks prepared by photolithography [Fig. 1(c)] served as paramagnetic controls.^27,28^ The dimensions of the Pd disks were chosen to match those of the a-GdCo disks (1.7 ± 0.1 *μ*m diameter; 30 ± 1.5 nm thick). This sample can be described as a magnetic system with a uniform, but varying, net magnetization, and the heating in this sample is expected to be accurately described by the uniform-magnetization-based theory. The heating should be close to zero as this is a paramagnet with no hysteresis loop area.

**FIG. 2.**
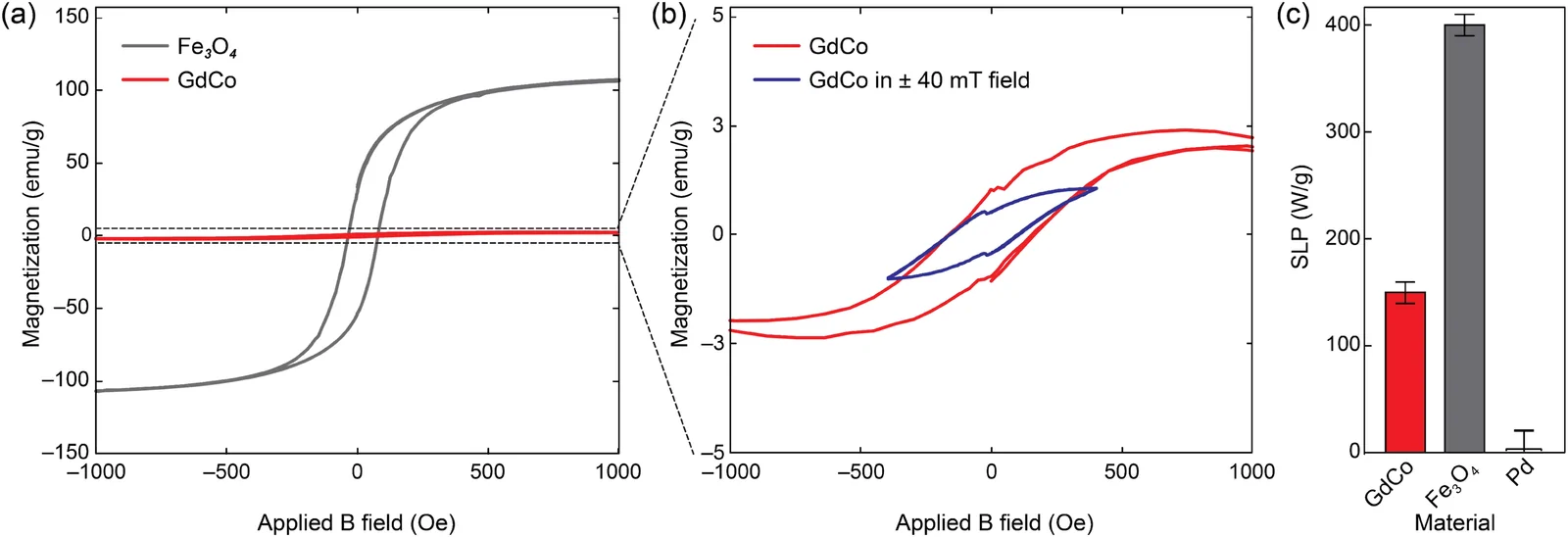
Experimentally measured properties of thermal nanotransducers. (a) SQUIDVSM hysteresis of a 29 nm diameter magnetite. Inset: the hysteresis of the GdCo to show relative magnitude of net magnetization. (b) SQUID-VSM hysteresis of GdCo, with the red loop showing the response of the system in a ±40 mT field. (c) Experimentally measured SLP at 165.3 kHz, 40 mT.

Vibrating sample magnetometry was performed on all three samples to measure the hysteresis loop area [Figs. 2(a), 2(b), and S3]. Hysteresis loops were also measured in a ±40 mT field, which is commensurate with the amplitude of the AMF used for heating experiments. The magnetite sample exhibited a hysteresis loop consistent with prior work^16,29^ with saturation magnetization of 105.0 emu/g_Fe_ [Fig. 2(a)]. The a-GdCo sample possessed a saturation magnetization of 2.7 emu/g_GdCo_, two orders of magnitude smaller than the magnetite sample, but still exhibited a measurable hysteresis loop area [Fig. 2(b)]. The Pd sample is a paramagnet^27,28^ and, therefore, exhibits a hysteresis loop with zero area (Fig. S3).

Specific loss power (SLP), heating efficiency in Watts per gram of active material,^30^ measurements were then performed on colloidal solutions of the above-mentioned samples in an AMF with an amplitude of 40 mT at 165.3 kHz (methods). The SLP was found to be 150 ± 20 w/g_GdCo_ for the a-GdCo sample, 400 ± 20 w/g_Fe_ for the magnetite sample, and 0 ± 40 w/g_Pd_ for the Pd sample [Fig. 2(c)]. Here, 40 mT sub-hysteresis loops are not explicitly shown for the magnetite sample as its ferromagnetic lattice saturates below 40 mT, or the Pd sample, as it is a paramagnet and does not exhibit hysteresis.

## III. DISCUSSION

### A. Uniform-magnetization heating theory

**FIG. 3.**
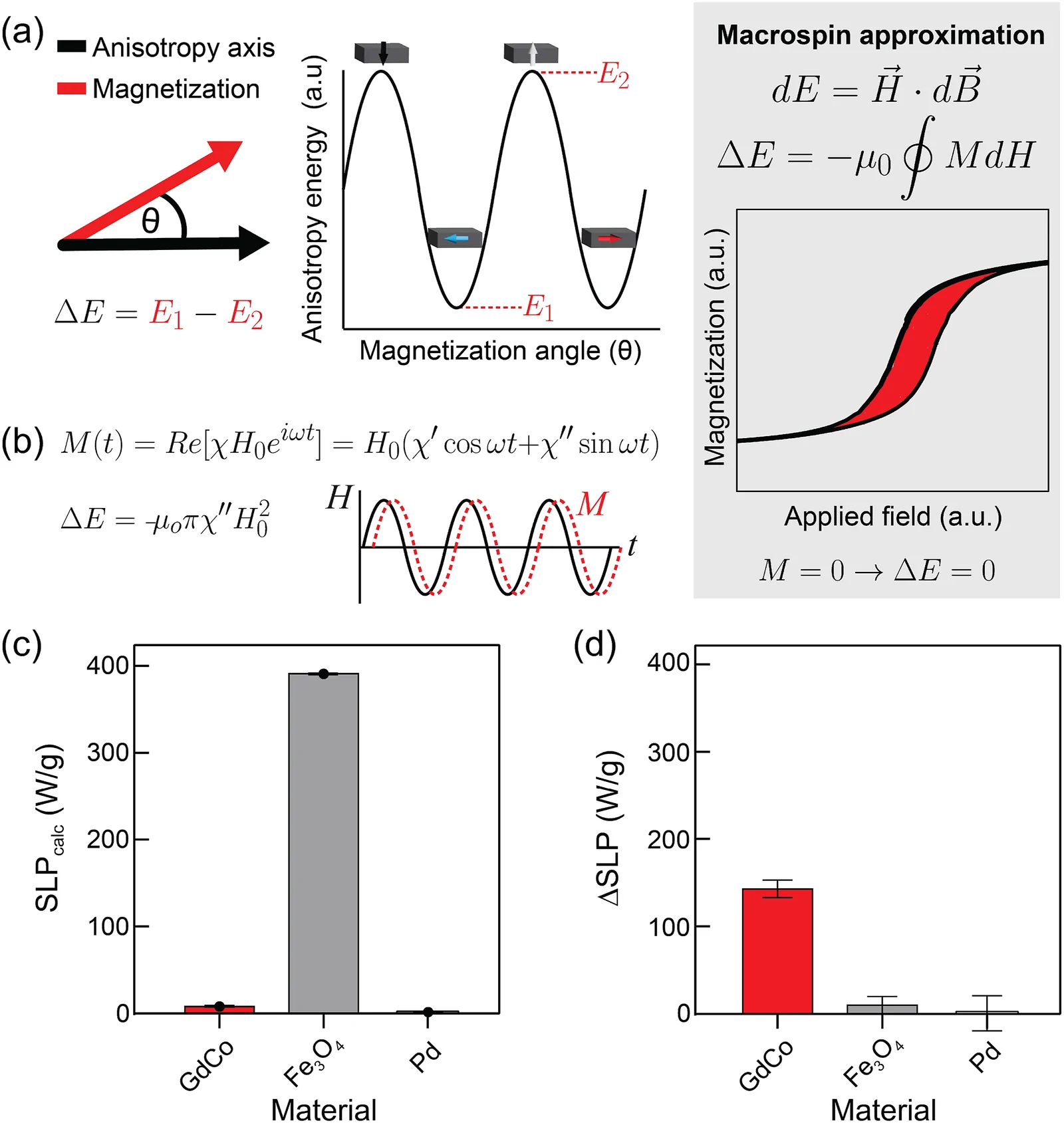
Uniform-magnetization-based AMF heating theory. (a) Dynamic hysteresis model that considers the energy losses associated with switching uniform magnetization over an anisotropy energy barrier. (b) Linear response theory that derives energetic losses from phase differences between an applied field and the driven magnetization. This relies on the assumption that magnetization is a linear function of the applied external field. Inset: For both models, energy loss is proportional to the hysteresis loop area and goes to zero when the magnetization goes to zero. (c) SLP calculated using the hysteresis loop area in a dynamic hysteresis model. The error bar values not visible on the graph are 0.001% for GdCo and Pd, and 0.2% for magnetite. (d) Difference between predicted heating using the dynamic hysteresis theory and experimentally measured heating.

In two commonly used theories that describe AMF-induced heating in magnetic materials, dynamic hysteresis and linear response theory,^15,16^ heating is derived from a simple physical model for a uniformly magnetized nanoparticle. In the dynamic hysteresis model, the reversal of uniform magnetization of the particles in an AMF and resultant heating is governed by an anisotropy energy barrier [Fig. 3(a)]. During this switching process, the particle’s anisotropy energy increases from a minimal value (*θ* = 0°, 180°) to a maximal value (*θ* = 90°, 270°) and returns to a low value [Fig. 3(a)]. The energy lost during the transition between the high- and low-energy states is assumed to be completely dissipated as thermal energy. The Hamiltonian for such a system with uniaxial anisotropy is

$$H=K_{\mathrm{eff}}Sin^{2}\theta -\vec{M}\cdot \vec{B},$$ (1)

where the first term is the anisotropy energy, *K*_*eff*_ is the effective anisotropy energy constant, and *θ* is the angle between the easy axis and the magnetization. The second term is the Zeeman energy, where $\vec{M}$ is the magnetization and $\vec{B}$ is the induction field.^16^ In this model, the total energy lost per cycle of AMF is equivalent to the area of the nanoparticles’ hysteresis loops [Figs. 3(a) and 3(b)]. This model offers insight into how increasing the magnitude of *M* and *K*_*eff*_ enhances heating from magnetic materials^16^ and offers design principles for magnetic multiplexing with thermal nanotransducers.^13,19^ The uniaxial magnetic dynamic hysteresis model should apply to all three systems, and we extracted our theoretical heating based on this approach. It applies to a-GdCo, even though it is amorphous, because it exhibits deposition-induced uniaxial anisotropy.^23,24^ The dynamic hysteresis model has also been extensively applied to magnetite nanoparticles.^6,13,19,29^ Although magnetite possesses cubic anisotropy, its behavior in an AMF applied along a single axis matches to the first order to that of a system with uniaxial anisotropy (see methods). The Pd sample acts as a null test case as both theories predict that paramagnetic Pd will not heat in a low-frequency AMF.

Another theory commonly used to describe AMF-induced heating is the linear response theory [Fig. 3(b)].^15^ In this model, magnetization of the particle is approximated with a linear function of the applied magnetic field, *M*(*H*) = Re[*χH*(*t*)], where *H*(*t*) = *He*^*iωt*^ and *χ* is the magnetic susceptibility of the material [Fig. 3(b)]. This theory does not describe the physical nature of energy loss in the magnetic nanoparticle, but derives heating due to out-of-phase components of magnetization switching when the particles are in an oscillating magnetic field. The out-of-phase component arises from the ferromagnetic nature of the system losing energy. Note that phase shifts in driven harmonically oscillating systems are due to energy dissipation, and similar principles are applied in atomic/magnetic force microscopy phase contrast imaging.^31,32^ While this model allows for variable magnetization, it still requires the magnetization to be uniform. In addition, the assumption that *M* depends linearly on *H* is only valid for a narrow range of systems^15,16^ and does not apply to the materials in this study for this given field and frequency range. As such, we do not experimentally investigate this here. However, under the conditions where AMF amplitude is low compared to the coercive field of the material, dynamic hysteresis and LRT are valid for the same materials.^16^ Under these conditions, LRT also predicts that heating is dependent on the area of the hysteresis loop and the net magnetization^16^ [Figs. 3(a) and 3(b)].

Although applicable to many common magnetic nanomaterials (e.g., magnetite nanoparticles), these models are not applicable for systems with non-uniform magnetization^8,17^ [Fig. 1(b)]. In addition, the energy terms employed in these models are also applicable to paramagnets, where a magnetic anisotropy term could, in principle, stem from shape anisotropy^33^ (Fig. S1). However, to observe appreciable heating in paramagnetic materials, the AMF frequency must be faster than individual spin dynamics since there is no exchange coupling. Typical paramagnets in AMFs with frequencies of 100 s of kHz to low MHz do not exhibit heating.^18,34^ Under conditions where paramagnetic systems exhibit large damping and, therefore, slow spin dynamics, heating can be measured at relatively low frequencies.^35–37^

As dynamic hysteresis is the appropriate model for the systems under investigation and AMF-induced heating is proportional to the hysteresis loop area swept by the net magnetization of the sample, we compared the hysteresis loop measurements for 29 nm magnetite, near-compensation a-GdCo microdisks, and paramagnetic Pd microdisks to experimentally measured heating (SLP).

The energy loss per cycle of AMF with an amplitude of 40 mT was calculated from the hysteresis loops for magnetite, a-GdCo, and Pd samples [Figs. 2(a), 2(b), and S3] and was found to be 2.38 mJ/g_Fe_, 0.049 mJ/g_GdCo_, and 0 J/g_*Pd*_, respectively. From the calculated energy loss per hysteresis loop cycle, an expected SLP for a 40 mT magnetic field at 165.3 kHz can be extracted. The calculated SLPs for the magnetite, GdCo, and Pd samples were 390 W/g_Fe_, 8 W/g_GdCo_, and 0 W/g_*Pd*_, respectively [Fig. 3(c)]. Although the measured [Fig. 2(c)] and calculated SLPs for magnetite and palladium are in good agreement [Fig. 3(d)], there is significant discrepancy between the calculated and measured SLP for GdCo [Fig. 3(d)], illustrating the limitations of dynamic hysteresis (and LRT) for predicting heating behavior of materials with non-uniform magnetization.

### B. Atomistic heating theory

**FIG. 4.**
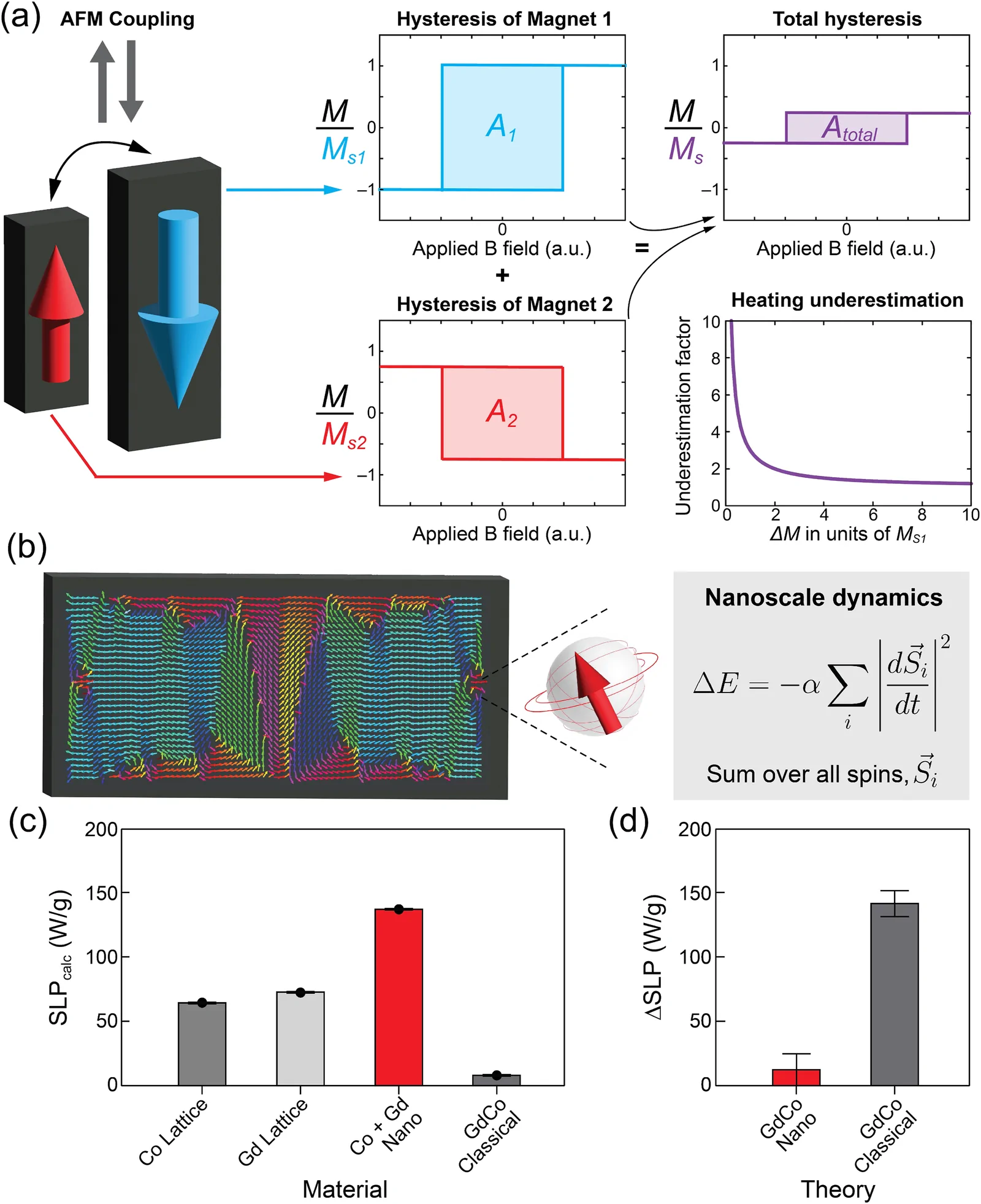
Nanoscale theory of heating. (a) Net heating of two antiferromagnetically coupled macroscopic ferromagnets in an alternating magnetic field is underestimated by a factor of (A_1_ + A_2_)/(A_total_). (b) Schematic of nanoscale dynamics model where energy losses are proportional to the magnitude of the velocity of each spin squared. In this model, uniform magnetization is not necessary to generate heating. (c) Calculated SLP extracted from the different sublattices of GdCo, their sum, and dynamic hysteresis theory heating prediction for GdCo. The error bar values not visible on the graph are 0.001% for GdCo and Pd, and 0.2% for magnetite. (d) Differences between experimentally measured and calculated SLPs for nanoscale theory of heating, and dynamic hysteresis theory of heating for a-GdCo.

To understand why the theory that relies on uniform magnetization cannot predict the thermal response of a-GdCo, we first consider a thought experiment involving two ferromagnets, *F*_1_ and *F*_2_, where $M_{s_{F1}}\cdot 0.75=M_{s_{F2}}$ that are antiferromagnetically coupled in an AMF [Fig. 4(a)]. This antiferromagnetic coupling could be realistic in the case where it is mediated by stray field interactions, such as observed in magnetic multilayers.^38^ In an AMF, the magnet with the larger magnetization will follow the applied field and the smaller magnet will be anti-aligned to the applied field [Fig. 4(a)], similar to what is observed in ferrimagnets [Fig. 1(b)]. Considering the net hysteresis loop area as a function of external field, the heating in this coupled non-uniform magnetic system would be underestimated by a factor of 7 based on the relative magnetizations of the two magnets [Fig. 4(a)], with the underestimation increasing as the magnetization of the two magnets equilibrates.

The fabricated a-GdCo ferrimagnets exhibit similar behavior to the above-mentioned thought experiment due to their non-uniform magnetization enforced via antiferromagnetic exchange [Fig. 1(b)]. To accurately recapitulate experimentally measured SLPs in GdCo, we need to consider individual spin dynamics and the contributions from both the Gd and Co lattices.

To understand the atomistic origins of heating in magnetic materials in an AMF, the behavior of individual magnetic spins should be considered [Figs. 4(a) and 4(b)]. Nanoscale spin dynamics have been calculated since 1954 using the Landau–Lifshitz–Gilbert (LLG) formalism,^39,40^ which is still used as the basis for most nanomagnetic simulations today.^41–43^ Simulations based on the LLG formalism have been extensively compared to experimental results and found to be accurate.^39^ The LLG formalism is based on the concept that each magnetic spin experiences an effective field, such that

$$\frac{d{\vec{S}}_{i}}{dt}=\frac{g\mu_{B}}{2\hbar }{\vec{S}}_{i}\times \mu_{0}{\vec{H}}_{\mathrm{eff}},$$ (2)

where^39,40^ ${\vec{S}}_{i}$ is the unit spin vector, such that $|{\vec{S}}_{i}|=1$ *ℏ*, and

$${\vec{M}}_{\mathrm{atom}}=\frac{-g\mu_{B}\mu_{u}}{2\hbar }{\vec{S}}_{i},$$ (3)

where ${\vec{M}}_{\mathrm{atom}}$ is the magnetic moment per atom, *g* is the spin g-factor, *μ*_*B*_ is the Bohr magneton, *μ*_*u*_ is the number of Bohr magnetons per atom, *μ*_0_ is the vacuum permeability, and *ℏ* is the reduced Planck constant. ${\vec{S}}_{i}$ will be referred to as the spin from this point on, and the gyromagnetic ratio will be defined as $\lambda =\frac{g\mu_{B}}{2\hbar }$.

${\vec{H}}_{\mathrm{eff}}$ contains appropriate versions of all micromagnetic energies and includes damping. An example of *H*_*eff*_ for spin *i* in a system involving symmetric exchange, anisotropy, and an external field can be given as

$$\begin{array}{ll} {\vec{H}}_{\mathrm{eff}} & =\left(\lambda {\vec{S}}_{i+1}+\lambda {\vec{S}}_{i-1}+\lambda AS_{i}^{x}{\vec{n}}_{x}+\lambda BS_{i}^{y}{\vec{n}}_{y}+\lambda CS_{i}^{z}{\vec{n}}_{z}+\mu_{0}\vec{H}\right)\\ & \;-\alpha (\lambda {\vec{S}}_{i+1}+\lambda {\vec{S}}_{i-1}+\lambda AS_{i}^{x}{\vec{n}}_{x}+\lambda BS_{i}^{y}{\vec{n}}_{y}\\ & \;+\lambda CS_{i}^{z}{\vec{n}}_{z}+\mu_{0}\vec{H})\times \lambda {\vec{S}}_{i}, \end{array}$$ (4)

where *A*, *B*, and *C* are anisotropy constants; ${\vec{n}}_{x,y,z}$ are unit vectors along *x*, *y*, and *z* axes; and *α* is the magnetic damping constant. This ${\vec{H}}_{\mathrm{eff}}$ accounts for anisotropy, symmetric exchange, and magnetic field energies and can be extended to include more exotic nanomagnetic energies not present in the systems under discussion, such as Dzyaloshinskii–Moriya interaction or spin–orbit coupling terms.^41,43^

In this description of magnetic systems, the energy losses are attributable to rapid movement of individual spins [Fig. 4(b)] and can be described as^40,44,45^

$$\frac{dE}{dt}=-\alpha \underset{i}{\sum }{\left|\frac{d{\vec{S}}_{i}}{dt}\right|}^{2}.$$ (5)

Ferromagnetic materials heat because of the rapid movement of spins induced by ferromagnetic exchange, whereas paramagnets do not heat because they are driven purely by the external magnetic field. More recently, an exact analytical solution for dynamic magnetic systems^45^ utilizing magnetic Feynman diagrams has also corroborated the energy losses predicted by applying the LLG to magnetic systems in Eq. (5).

The atomistic approach also explains why ferromagnets heat in AMFs and paramagnets do not; direct exchange induces rapid switching of spins. Paramagnets do exhibit damping, as utilized in magnetic resonance imaging,^46^ so in theory, they should noticeably heat if driven by a saturating field that induces spin switching on the same time scale as ferromagnetic exchange induces switching. Exchange-induced switching occurs on the order of nanoseconds for ferromagnets. Consequently, for paramagnetic heating to be observable, the applied AMF should have a frequency in the GHz range, in addition to fully saturating the sample.

Finally, for a single uniformly magnetized system (e.g., an individual spin or a macrospin), the energy loss described by Eq. (5) is proportional to the area swept by the hysteresis loop in its effective field.^44^ Equivalently, the hysteresis loop area of a magnetic system can be calculated from LLG dynamics and used to approximate the heating of a uniformly magnetized material. In summary, magnetic heating in an AMF can be accurately calculated by considering the hysteresis loop swept by each individual spin as a function of *H*_*eff*_, rather than the net hysteresis loop of the system. This heating can also be calculated by integrating Eq. (5) over the time it takes for the switching process induced by the external field.

To explain the experimental discrepancy observed between classical heating theory and the heating measured in a-GdCo [Fig. 3(d)], the *H*_*eff*_ model and the nanoscale heating dynamics model can be combined to extract predicted heating contributions from the Gd and Co sublattices. The calculations in the following rely on two physical assumptions: (1) the Gd and Co sublattices are uniformly magnetized, and (2) the spins of the Gd and Co lattices are locked in an antiparallel arrangement. These assumptions are supported experimentally^22–24,47–49^ and theoretically with atomistic simulations (Fig. 1(b); supplementary material, Fig. S2).

In these near-compensation GdCo microdisks, Gd forms the majority lattice. We note that the magnetization of Gd is a uniform quantity and Gd–Co coupling can be ignored because the two sublattices are “locked” relative to each other, negating any change in this energy term during switching. As a result, the *H*_*eff*_ field acting on this system is the externally applied field *H*_*external*_. The *H*_*eff*_ field produced by the Co sublattice on the Gd sublattice points in the direction opposite to the Gd sublattice magnetization. Consequently, it is aligned with the anisotropy axis and anti-aligned with the applied external field when the Gd lattice is in an anisotropy energy minima. Therefore, the effect of Co on the Gd lattice can be viewed as an increase in the anisotropy energy term. Indeed, the increase in the effective anisotropy in GdCo as it approaches compensation has been experimentally observed.^22,23^

Since the Co sublattice dynamics are dominated by ferrimagnetic exchange, rather than the external field, it is more challenging to calculate the energy losses in this sublattice. To circumvent this problem, spin dynamics will be used.

The Gd and Co sublattices can be treated as uniform magnets,

$$\frac{d{\vec{S}}_{Co,i}}{dt}=\frac{d{\vec{S}}_{Co,j}}{dt}\;\text{and}\;\frac{d{\vec{S}}_{Gd,i}}{dt}=\frac{d{\vec{S}}_{Gd,j}}{dt},\;\;\forall i,j,$$ (6)

where ${\vec{S}}_{Co,j}$ and ${\vec{S}}_{Gd,i}$ are spins in the Co and Gd lattices, respectively. The physical assumption that the Gd and Co lattices are locked opposite each other gives

$$\frac{d{\vec{S}}_{Gd,i}}{dt}=-\frac{d{\vec{S}}_{Co,j}}{dt},\;\;\forall i,j.$$ (7)

Matching saturation magnetization with the total number of atoms for each lattice *N*_*a*_ and using Eq. (3) then returns

$$\frac{M_{s_{Gd}}}{M_{s_{Co}}}=\frac{N_{a_{Gd}}\mu_{u_{Gd}}}{N_{a_{Co}}\mu_{u_{Co}}}.$$ (8)

In addition, since heating due to energy losses must match across theories for Gd (and Gd is the majority lattice),^44^

$$\Delta E_{\tau_{Gd}}=-A_{Gd}=-\alpha \overset{\tau }{\underset{0}{\int }}\underset{i}{\sum }{\left|\frac{d{\vec{S}}_{i_{Gd}}}{dt}\right|}^{2}dt,$$ (9)

with *A*_*Gd*_ denoting the area of the Gd hysteresis loop and where *τ* is the period of the AMF ($\tau =\frac{1}{f}$, where f is the AMF frequency).

We define the nanomagnetic heating definition of energy loss from a single AMF cycle, Δ*E*_*τ*_, in the Co lattice as

$$\Delta E_{\tau_{Co}}=-\alpha \overset{\tau }{\underset{0}{\int }}\underset{i}{\sum }{\left|\frac{d{\vec{S}}_{i_{Co}}}{dt}\right|}^{2}dt.$$ (10)

Using Eqs. (6) and (7),

$$\Delta E_{\tau_{Co}}=-\alpha \overset{\tau }{\underset{0}{\int }}N_{a_{Co}}\mu_{u_{Co}}{\left|\frac{d{\vec{S}}_{Co}}{dt}\right|}^{2}dt$$ (11)

and using Eq. (8),

$$\begin{array}{ll} \Delta E_{\tau_{Co}} & =-\alpha \overset{\tau }{\underset{0}{\int }}\frac{M_{s_{Co}}}{M_{s_{Gd}}}N_{a_{Gd}}\mu_{u_{Gd}}{\left|\frac{d{\vec{S}}_{Co}}{dt}\right|}^{2}dt\\ & =-\alpha \overset{\tau }{\underset{0}{\int }}\frac{M_{s_{Co}}}{M_{s_{Gd}}}N_{a_{Gd}}\mu_{u_{Gd}}{\left|\frac{d{\vec{S}}_{Gd}}{dt}\right|}^{2}dt, \end{array}$$ (12)

which yields

$$\Delta E_{\tau_{Co}}=-\frac{M_{s_{Co}}}{M_{s_{Gd}}}\alpha \overset{\tau }{\underset{0}{\int }}N_{a_{Gd}}\mu_{u_{Gd}}{\left|\frac{d{\vec{S}}_{Gd}}{dt}\right|}^{2}dt=-A_{Gd}\frac{M_{s_{Co}}}{M_{s_{Gd}}}.$$ (13)

The energy losses in both sublattices can then be calculated from the hysteresis loop area of the Gd sublattice. This information can be extracted from the net hysteresis loop of GdCo [Fig. 2(b)] by recognizing that the Gd and Co sublattice magnetizations are proportional to the ratio of atomic percentages multiplied by atomic spins,

$$\frac{M_{s_{Co}}}{M_{s_{Gd}}}=\frac{Co_{N_{s}}\cdot 0.75}{Gd_{N_{s}}\cdot 0.25},$$ (14)

and that the total magnetization of the GdCo alloy is the sum of the Gd and Co sublattice magnetization,

$$M_{s_{Gd}}-M_{s_{Co}}=M_{s_{\mathrm{Total}}}.$$ (15)

Using experimentally determined *M*_*s*_ = 2.7emu/g_GdCo_ [Fig. 2(b), red loop], $M_{s_{Gd}}$ and $M_{s_{Co}}$ are found to be 24.6 and 21.8 emu/g_GdCo_, respectively. Using these values, one can then determine energy loss per cycle of AMF for the Gd and Co sublattices to be 0.44 and 0.39 mJ, respectively. Multiplying by the AFM frequency in our measurement setup, we obtain a calculated SLP of 72.9 W/g_GdCo_ from the Gd sublattice and 64.6 W/g_GdCo_ from the Co sublattice [Fig. 4(c)]. Adding these losses together, we obtain an SLP of 137.5 W/g_GdCo_ [Fig. 4(c)]. Calculated using spin dynamics, this value agrees with experimental observations, unlike the values obtained from heating theories developed for uniform magnets [Fig. 4(d)].

## IV. CONCLUSIONS

Heat dissipation in an AMF was measured in three magnetically distinct colloidal particle samples: uniformly magnetized magnetite, near-compensation amorphous GdCo, and paramagnetic Pd. Theories that rely on the assumption that the magnetization in the material is a uniform quantity are able to explain the heating of magnetite and Pd, as this physical assumption is valid for these two systems. However, in near-compensated ferrimagnetic Gd_25_Co_75_, there is more than an order-of-magnitude discrepancy between the heating predicted by uniform-magnetization-based theories and the heating observed in GdCo [Figs. 3(c) and 3(d)]. This is due to the invalid assumption of a homogeneous magnetic system in GdCo, which has an active magnetic lattice that is non-uniform at the atomic scale. To explain heating in GdCo, we employed a model incorporating spin dynamics. In this model, heating is driven by the rapid movement of individual spins rather than a net magnetization reversal for the entire particle. The heating of the GdCo sublattices was extracted from the macroscopic magnetic properties of this material and was found be to within error of the measured experimental values [Figs. 4(c) and 4(d)].

Since the a-GdCo microdisks have a cross-sectional surface area 3000 times larger than the magnetite nanoparticles, it is conceivable that some heating in an AMF could be generated by eddy currents. Pd microdisks with dimensions matching those of a-GdCo disks act as an upper bound for the possible eddy current heating as their resistivity is 20 times lower than that of GdCo.^50^ As no heating is found for Pd microdisks, the effects of eddy currents can be ignored for GdCo as well. We note that, excluding the magnetic structure, this is the only expected difference between the differently sized systems in this AMF heating context.

This model provides physical insight into the atomistic origin of magnetic heating: AMF-induced energy loss requires rapid exchange-driven movement of spins, which suggests optimization strategies for magnetothermal nanotransducers, or any system with a non-uniform magnetization. The results from the atomistic approach are not surprising in light of the well-established LLG formalism. Future generations of heat-dissipating nanomaterials will likely invite further examination of non-collinear magnetic systems, such as magnetic vortices^51^ or skyrmions, where energy loss is underestimated by traditional uniform-magnetization models. This paper does not address these non-collinear structures driven by multiple types of exchange coupling. It remains an open and non-trivial theoretical challenge to connect the spin dynamics of these more complex, non-collinear systems with their macroscopic magnetic properties and heating.

## SUPPLEMENTARY MATERIAL

The supplementary material figure file contains figures showing additional simulation results and experimental data. The supplementary material methods file contains experimental details, additional notes on the magnetic simulations, and mathematical details. The supplementary material file Vampire_GdCo_Magnetite.zip contains the input and material files for VAMPIRE atomistic magnetic simulations.

## Data Availability

The data that support the findings of this study are available from the corresponding author upon reasonable request.
